# Characterizing *in vivo* loss of virulence of an HN878 *Mycobacterium tuberculosis* isolate from a genetic duplication event

**DOI:** 10.1016/j.tube.2022.102272

**Published:** 2022-11-02

**Authors:** Bryan J. Berube, Sasha E. Larsen, Matthew B. McNeil, Valerie A. Reese, Tiffany Pecor, Suhavi Kaur, Tanya Parish, Susan L. Baldwin, Rhea N. Coler

**Affiliations:** a Seattle Children’s Research Institute, Center for Global Infectious Disease Research, Seattle, WA, USA; b TB Discovery Research, Infectious Disease Research Institute, Seattle, WA, USA; c Department of Pediatrics, University of Washington School of Medicine, Seattle, WA, USA; d Department of Global Health, University of Washington, Seattle, WA, USA

**Keywords:** *Mycobacterium tuberculosis*, Genetic duplication, HN878, Virulence, Mouse model

## Abstract

The increase of global cases of drug resistant (DR) *Mycobacterium tuberculosis* (M.tb) is a serious problem for the tuberculosis research community and the goals to END TB by 2030. Due to the need for advancing and screening next generation therapeutics and vaccines, we aimed to design preclinical DR models of Beijing lineage M.tb HN878 strain in different mouse backgrounds. We found escalating sensitivities of morbidity due to low dose aerosol challenge (50–100 bacilli) in CB6F1, C57BL/6 and SWR mice, respectively. We also observed that pulmonary bacterial burden at morbidity endpoints correlated inversely with survival over time between mouse strains. Interestingly, with *in vitro* passaging and in the process of selecting individual DR mutant colonies, we observed a significant decrease in *in vivo* HN878 strain virulence, which correlated with the acquisition of a large genetic duplication. We confirmed that low passage infection stocks with no or low prevalence of the duplication, including stocks directly acquired from the BEI resources biorepository, retained virulence, measured by morbidity over time. These data help confirm previous reports and emphasize the importance of monitoring virulence and stock fidelity.

## 1. Introduction

An alarming increase in the total number of deaths from tuberculosis (TB) and cases of drug resistant (DR) *Mycobacterium tuberculosis* (M.tb) was noted for 2020 in the most recent World Health Organization Global TB report [1]. This included a ten percent increase in the global detection of front line drug rifampicin resistance in microbiologically confirmed pulmonary TB [1], up to 71% of total cases in 2020. These two monitoring criteria, drug resistance and deaths due to TB, are intimately tied to disruptions in care, case finding, and diagnosis over the past years of the COVID-19 pandemic [2,3]. While these are disappointing after a decade of gains for TB care and case finding, the marked increase in detection of DR-M.tb is partially due to increased awareness and screening for DR in 2020 compared to 2019 [1]. The enhanced detection of DR-M.tb has led to expanded coverage of novel drug regimens for drug resistant infections.

Due to the considerable acceleration of DR-M.tb, we and others [4-6] have set out to develop preclinical models of DR M.tb emergence to test novel and adjunctive treatments, including therapeutic vaccinations [7]. Indeed, fit-for-purpose preclinical models that reflect specific global populations was a key benchmark identified in the Global Roadmap for Research and Development of Tuberculosis Vaccines [8]. Many aspects contribute to preclinical model design and application [9,10]. In the context of modeling TB in the mouse, host genetics [11-13], challenge dose [14] and M.tb isolate [15] are all key variables to consider modifying depending on the study goals. For example, selection of virulent and diverse clinical M.tb isolates representative of specific geographic regions [16,17] may directly influence the translational potential of a given model. We hypothesize that lineage prevalence in different regions may shape things like vaccine efficacy due to differential host gene expression as seen in the proteomic signatures profiled by Yimer et al., in 2020 [18].

Due to the enhanced acquisition of DR in the M.tb Beijing Lineage[19] and its expanding global distribution [20], we prioritized a Lineage 2 isolate for this work. Representative clinical isolate M.tb HN878 was isolated from a 1990s outbreak in Houston, Texas [21] and has its full genome sequenced (GenBank: ADNF01000000.1). M.tb HN878 is considered a hypervirulent isolate which may directly affect TB disease phenotypes and pathology in preclinical models, as was recently demonstrated in a mouse model showing HN878 induces more granuloma-like structures than the lab-adapted H37Rv [21,22]. Interestingly HN878 also has a documented propensity for genetic duplications under selective pressure [23]. In this work, we describe the development of preclinical mouse models across three genetic backgrounds (SWR – highly susceptible, C57BL/6 – moderately susceptible, and CB6F1 – moderately resistant) using DR M.tb HN878 strains and characterize a loss of virulence with genetic duplication after multiple passages of HN878 *in vitro*.

## 2. Results

**Fig. 1.**
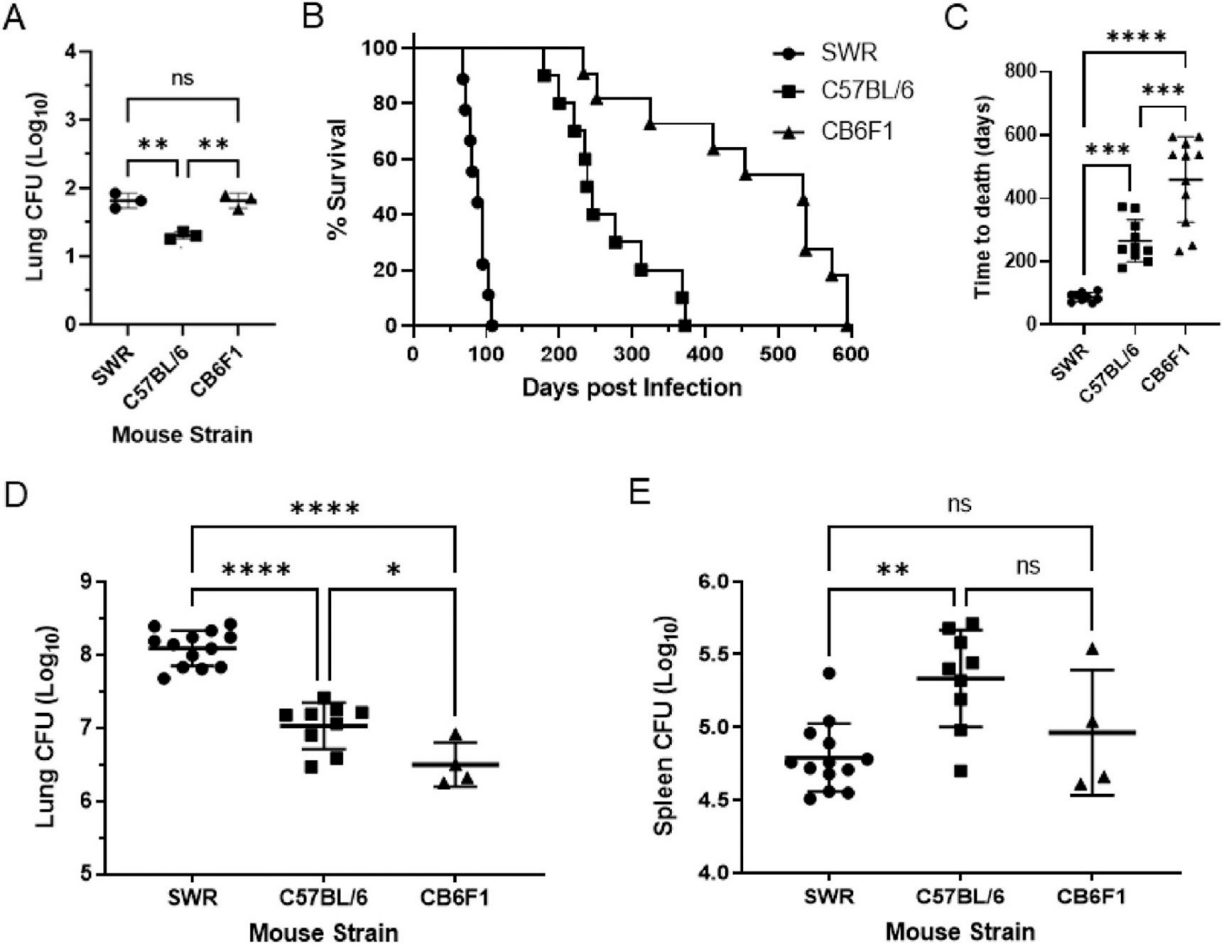
Characterizing M.tb HN878 virulence across three mouse strains. Cohorts of SWR, C57BL/6, or CB6F1 mice were infected with HN878. P1. (A) Bacterial burden was measured in the lungs at 24 h post-infection to monitor initial lung deposition. (B–C) Mice were monitored for survival over time. Bacterial burden was measured in both the lungs (D) and spleen (E) at the time of euthanasia for each mouse. Infection of the three mouse strains were carried out on different days, but are compared on the same graphs. ANOVA with Tukey’s post hoc test was used to compare groups, statistically significant differences are denoted by asterisk.

To establish preclinical animal models of HN878 infection for use in future vaccine and drug development studies, we infected three different mouse strains with a low dose aerosol (LDA) infection of HN878. While CFU deposited in the lung at 24 h post-infection were nearly identical for SWR (mean CFU 66.7, n = 3) and CB6F1 mice (mean CFU 66.3, n = 3), with just slightly decreased counts in C56BL/6 mice (mean CFU 20.3, n = 3) (Fig. 1A), the three mouse strains displayed drastically different susceptibilities to HN878 (Fig. 1B). SWR mice were highly susceptible to HN878 with an average survival of only 87 days. C57BL/6 mice had an intermediate susceptibility with an average survival of 265 days, while CB6F1 were relatively resistant to HN878 infection with an average survival of 459 days, significantly longer than the other two mouse strains (Fig. 1C). Susceptibility of individual mouse strains to HN878 was significantly correlated with lung bacterial burden at time of euthanasia; there was an inverse relationship between CFU at time of euthanasia and average time to death, with SWR mice having the largest (mean Log10 CFU 8.1, n = 13) and CB6F1 mice the smallest lung bacterial loads (mean Log10 CFU 6.5, n = 4), respectively (Fig. 1D). There was no correlation with susceptibility and CFU in the spleen, as C56BL/6 mice had slightly higher bacterial loads (mean Log10 CFU 5.3, n = 9) than SWR mice (mean Log10 CFU 4.8, n = 13) (Fig. 1E), suggesting increased dissemination of HN878 was not responsible for the enhanced morbidity observed in SWR mice.

**Fig. 2.**
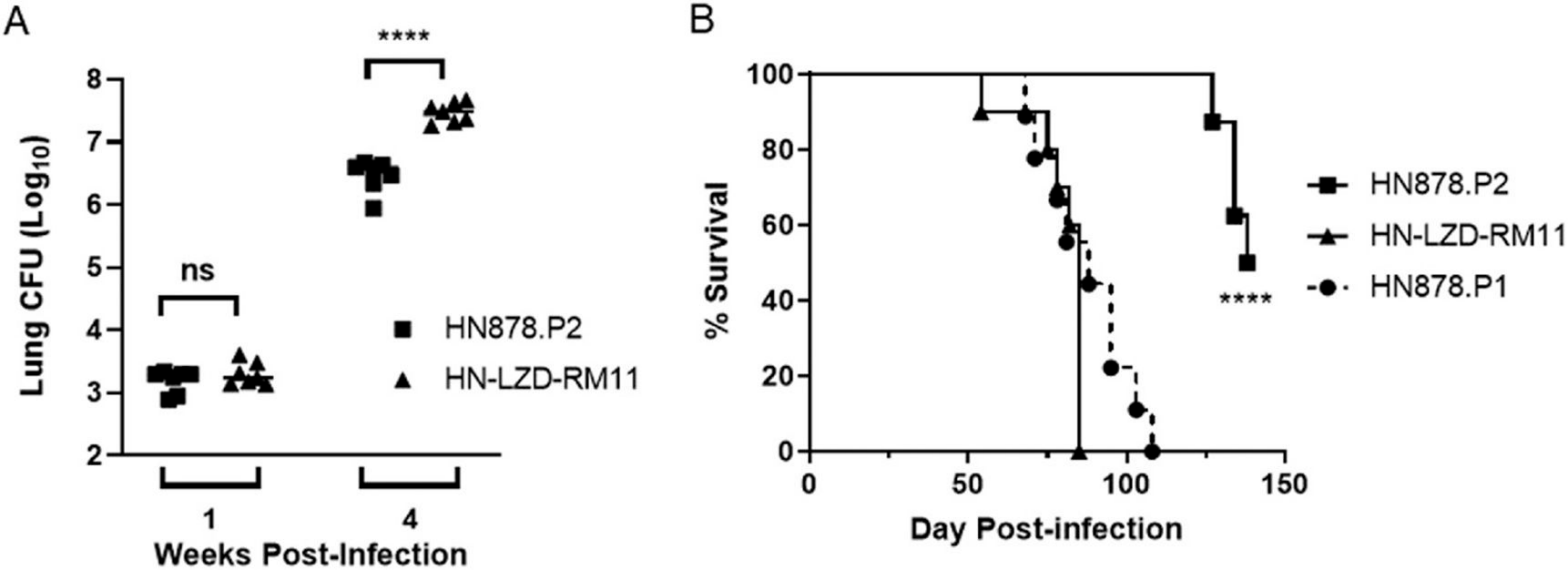
Lost *in vivo* virulence of passaged HN878 M.tb in SWR mice. Cohorts of SWR mice were infected with LDA HN878.P2 or HN-LZD-RM11. (A) Groups of mice (n = 7) were assessed for bacterial burden at 1 and 4 weeks post-infection. **** indicates p value < 0.0001; ns = not significant as measured by a two-tailed *t*-test. (B) Groups of mice (n = 10) were monitored for survival. **** indicates a statistically significant difference (p < 0.0001) between HN878. P2 and HN-LZD-RM11 groups as measured by a Mantel-Cox log-rank test. Survival curve of mice infected with HN878.P1 from Fig. 1A is shown for comparison (dashed line).

We next tried to establish a mouse model of drug-resistant infection in SWR mice by challenging mice with wild-type HN878 or a lab-generated strain resistant to the second-line TB drug, linezolid (HN-LZD-RM11). As our parent HN878 infection stocks (hereafter referred to as HN878 Passage 1 or HN878.P1) had been depleted for other studies, we passaged the bacteria and created a new bank of HN878 infection stocks referred to as HN878 Passage 2 or HN878.P2. Upon infection of SWR mice, HN878.P2 and HN-LZD-RM11 both established infection and replicated to equivalent bacterial loads (HN878.P2 mean Log10 CFU 3.2, n = 7; HN-LZD-RM11 mean Log10 CFU 3.3, n = 7) in the lung by one-week post-infection (Fig. 2A). However, by week 4, HN-LZD-RM11 (mean Log10 CFU 7.5, n = 3) had replicated to higher bacterial burden than HN878.P2 (mean Log10 CFU 6.5, n = 3, Fig. 2A) leading to enhanced morbidity and mortality (Fig. 2B). Surprisingly, while survival curves for HN-LZD-RM11 were nearly identical to those in Fig. 1 with HN878.P1 (dashed line in Fig. 2B), HN878.P2-infected mice were significantly delayed in time to death (Fig. 2B). The average survival time for the HN-LZ-RM11 and HN878.P1 infected cohorts were 85 and 88 days, respectively, whereas HN878.P2 was 138 days, a difference of 53 days compared to HN878.P1 (Fig. 2B).

**Fig. 3.**
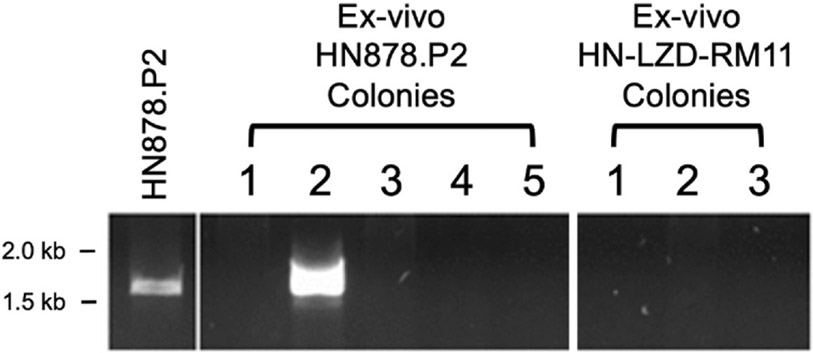
Passaged M.tb HN878.P2 contains genetic duplication event in a proportion of the stock. Individual colonies (n = 5 per strain) isolated from *ex vivo* lung homogenates were assessed for the presence of a duplication event known to occur in HN878 isolates [23]. Samples were compared to bulk DNA isolated from infection stocks prepared for HN878.P2 (left).

A previous report identified the existence of a duplication event in the HN878 strain, which arises upon successive passaging of the strain *in vitro* [23]. Given the reduced virulence in morbidity seen with HN878. P2, we purified DNA from colonies isolated from the lungs of euthanized mice (Fig. 2B). While none of the colonies isolated from HN-LZD-RM11 contained the duplication, one in five colonies from HN878.P2-infected mice was positive for the duplication (expected PCR band of ~1.6 kb), as was bulk DNA isolated from the HN878.P2 infection stock used for the LDA challenge (Fig. 3). The fact that one in five colonies from HN878. P2-infected mice contained the duplication suggests that either the duplication event reverts over time *in vivo* (as suggested previously [24]), or the infection stock is a mixed population with only a portion of the bacteria containing the duplication.

**Fig. 4.**
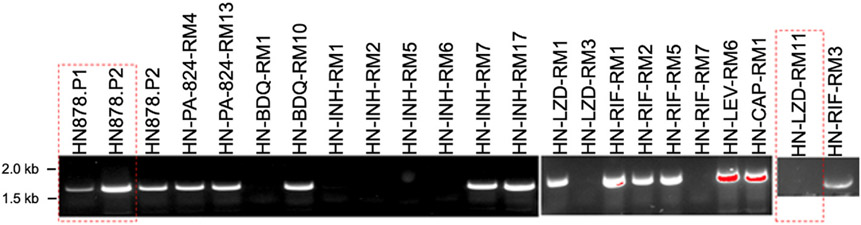
High frequency of genetic duplication in laboratory-generated mono-resistant strains. Bulk DNA from infection stocks of a panel of laboratory-generated resistance mutants were tested by colony PCR for the presence of the HN878 duplication and compared to the HN878.P1 and HN878.P2 infection stocks. Isolates outlined in red dashed rectangles are those tested *in vivo* in this study.

As all our lab-generated resistant mutants are grown from a single colony, we tested a set of 20 HN878 clones we had generated to be resistant to a panel of first- and second-line drugs used to treat Mtb (PA-824, Bedaquiline (BDQ), Isoniazid (INH), Linezolid (LZD), Rifampicin (RIF), Levofloxacin (LEV), Capreomycin (CAP)). The HN878.P2 infection stock and 12/20 resistant mutants tested positive for the duplication event by PCR, while HN-LZD-RM11 and 7 other resistant mutants did not (Fig. 4; all samples were positive by PCR for a control gene (data not shown)). These data are consistent with the hypothesis that the reduced virulence of HN878.P2 seen in Fig. 2 could be due to a duplication event in the infection stock. HN878.P1 did also present positive for the duplication (Fig. 4). However, the PCR reaction is qualitative, not quantitative, and we hypothesize there may be a difference in abundance of the duplication between the two stocks, such that the frequency of the duplication in HN878.P1 is below a certain threshold so as to not affect virulence.

**Fig. 5.**
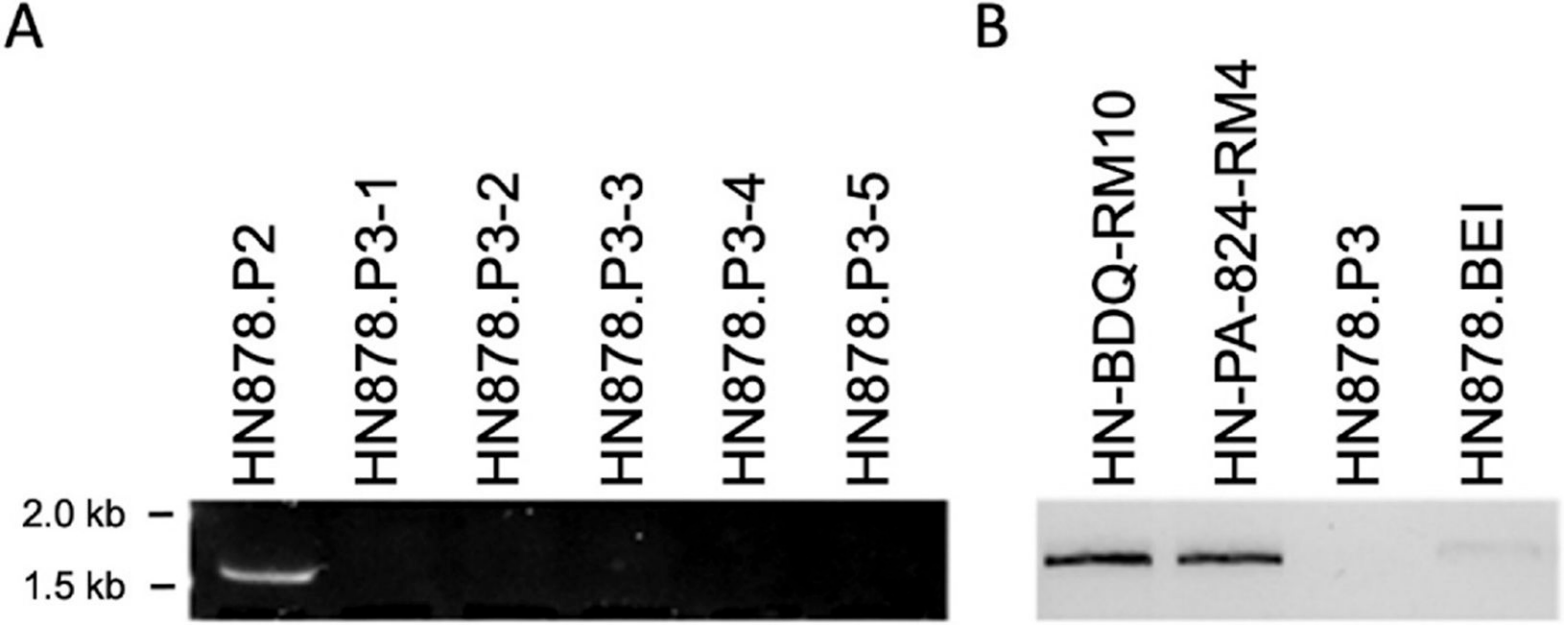
Low passage M.tb HN878 demonstrates lower frequency of genetic duplication. Bulk DNA was prepared from 5 different stocks of HN878.P3 (A) and tested by colony PCR for the HN878 duplication. DNA samples were compared to the HN878.P2 (B) infection stock as well as HN878-BEI isolate.

To further support this hypothesis, we isolated individual colonies from HN878.P2 and selected 5 lacking the duplication event (Fig. 5A). We expanded one of the colonies lacking the duplication to create a new infection stock (HN878.P3). In parallel, we ordered and expanded an HN878 isolate from BEI. In both cases, we took care to limit the number of passages before freezing. When tested for the duplication event, the HN878.BEI infection stock showed a very faint band (Fig. 5B), suggesting the duplication event is present in a subset of bacilli, but at a very low abundance. HN878.P3 did not show any detectable sign of the duplication (Fig. 5B).

**Fig. 6.**
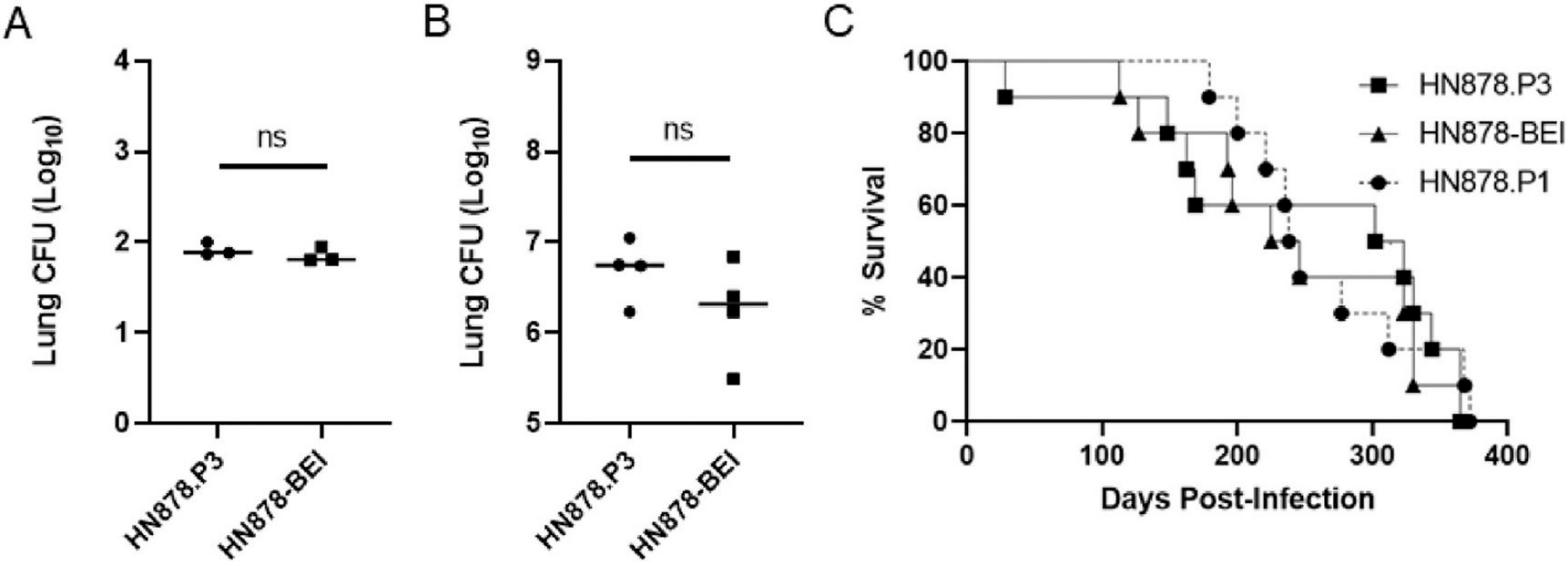
Low passage M.tb HN878 without high frequency genetic duplication retains high virulence in C57BL/6 mice. Cohorts of C57BL/6 mice were infected with HN878.P3 or HN878-BEI. Groups of mice (n = 7) were assessed for lung bacterial burden at 1 (A; n = 3) and 4 weeks post-infection (B; n = 4). (C) Groups of mice were monitored for survival. There was no significant difference between HN878.P3 and HN878-BEI groups as measured by a Mantel-Cox log-rank test. Survival curve of mice infected with HN878.P1 (dash-circles) from Fig. 1A is shown for comparison. ns = not significant.

We next tested virulence of the HN878.P3 and HN878.BEI infection stocks in a C57BL/6 mouse model of survival. C57BL/6 mice were leveraged here as they have a longer survival time (average 265 days) than the previously used SWR mice (average 87 days) and this affords greater resolution of differences in M.tb virulence being assessed. Lung CFUs were identical between the two groups at 1-week post-infection (HN878.P3 mean Log10 CFU 1.91, HN878.BEI mean Log10 CFU 1.85 n = 3; Fig. 6A). While there was a slight trend towards increased bacterial burden with HN878.P3 (mean Log10 CFU 6.63) over HN878.BEI (mean Log10 CFU 6.60, n = 4) at 4 weeks post-infection (Fig. 6B), the differences were not significant. Both isolates caused significant morbidity and mortality starting between 100 and 200 days post-infection with no significant differences between the two groups (median survival HN878.P3 312.5 days, HN878.BEI 235.5 days; p = 0.5812 Mantel-Cox log-rank test) or compared to the previous HN878.P1 infection in C57BL/6 mice (Fig. 6C). Indeed, both stocks observed their final mortality endpoint on day 330 post-challenge (Fig. 6C).

## 3. Discussion

Here we profiled escalating sensitivity to M.tb infection with Beijing isolate HN878 across three mouse strains (CB6F1 < C57BL/6 < SWR) where an equal infectious challenge resulted in disparate morbidity and mortality over time. We also found that the pulmonary bacterial burden inversely correlated with time to morbidity endpoints. These data are consistent with previous studies using laboratory strains of M.tb showing variable susceptibility to infection in different mouse models with SWR mice being highly susceptible to infection (PMID:9616378; PMID:12933873). Our data confirms an inherent susceptibility of different mouse strains to M.tb infection regardless of whether a laboratory or clinical isolate is used for infection and highlights the importance of selecting an appropriate mouse model for drug development and vaccine studies. More data needs to be generated across multiple animal species and human clinical trials to model which mouse species (if any) will more accurately predict outcomes of future clinical trials.

We were originally developing these animal models as a tool to characterize the emergence of DR *in vivo* across mouse strains using lab-developed mono-DR mutants. During the development, we observed a significant loss of virulence *in vivo* from HN878 strains which had been passaged *in vitro* for expansion of stocks or selection for DR mutations. Using colony PCR techniques reported in the literature^23^, we found that those strains with reduced virulence also harbored a genetic duplication event known to significantly impact virulence (PMID:24778110; PMID:20639330). Interestingly, a significant fraction of *ex vivo* colonies isolated from mouse lung homogenates plated on agar plates for enumeration did not harbor the duplication. The fitness gained by the duplication for *in vitro* growth seems dispensable for *in vivo* persistence. However, the large presence of the genetic duplication at challenge, seems sufficient to dramatically affect morbidity in mouse models. In these studies, we leveraged a standard or LDA (50–100 CFU at challenge) which likely tolerates a higher background of duplication than an ultra-low dose (ULDA 1–10 CFU) challenge model. We hypothesize that in an ULDA challenge the proportion of bacteria harboring the genetic duplication could vary substantially. In the context of an ULDA challenge where mice are infected with a single CFU, the presence or absence of the duplication event in that single CFU could drastically influence infection outcomes. Collectively these data confirm earlier reports [23, 24] and impress the importance of strain and stock maintenance for *in vivo* preclinical modeling.

## 4. Materials & methods

### HN878 isolates.

*M.tb* HN878 was a gift from Ian Orme (Colorado State University, Fort Collins, CO). HN878.P1 was prepared by growing and propagating the original stock in 7H9-Tw-OADC (Middlebrook 7H9 media containing 0.05% (w/v) Tween-80 and 10% (v/v) Middlebrook oleic acid, albumin, dextrose, catalase (OADC) supplement) under aerobic conditions. Cultures were expanded and frozen at −80 °C in 25% glycerol. To prepare HN878.P2 infection stocks, HN878.P1 was serially passaged and expanded in 7H9-Tw-OADC to an optical density (OD_600_) of 1.0. The bacterial culture was mixed 1:1 with 50% (w/v) glycerol and aliquoted and frozen at −80 °C. To prepare HN878.P3, individual colonies of HN878.P2 were assessed by colony PCR, as described below. Colonies lacking the duplication event were expanded with the least number of passages possible and frozen as with HN878.P2. HN878.BEI was ordered from BEI Resources (NR-13647; Manassas, VA), propagated in 7H9-Tw-OADC with a minimum number of passages, and frozen as above. M.tb DR mutants were prepared by plating HN878.P1 onto agar plates containing 5X the minimum-inhibitory concentration of each individual drug. Individual colonies were expanded and drug-resistance confirmed by standard minimum inhibitory concentration growth assays, as described previously [25].

### Preclinical Animal Models.

Female SWR/J mice 4–6 weeks of age were purchased from Jackson Laboratory, and 4–6 week old female C57BL/6 and female CB6F1 mice were purchased from Charles River Laboratory. Mice were housed under pathogen-free conditions at either at Seattle Children’s Research Institute (SCRI) or the Infectious Disease Research Institute (IDRI) biosafety level 3 animal facility and were handled according to the guidelines of each Institutional Animal Care and Use Committee. Mice were challenged with low dose (50–100 bacteria) aerosols (LDA) of various passaged and isolates of M.tb HN878 using a University of Wisconsin-Madison aerosol chamber or Glas-Col whole body aerosol chamber.

### Bacterial Burden Assessment

24 h to 4 weeks post infection with M.tb, 3–7 mice per group were humanely euthanized with CO_2_. Lung and spleen tissue from infected animals were isolated and homogenized in 5 mL PBS + Tween-80 (Sigma-Aldrich, St. Louis, Missouri, USA) CFU buffer using an Omni tissue homogenizer (Omni International, Kennesaw, GA, USA). Tissue homogenates were serially diluted in CFU buffer and plated on Middlebrook 7H10 agar plates. Plates were incubated at 37 °C and with or without 5% CO_2_ for 2–3 weeks before colonies were counted. Bacterial burden, as CFU/mL, was calculated per organ and is presented as Log10 values.

### Survival.

Cohorts of 10 mice were identified and weighed regularly post challenge for survival assessments. Mice were euthanized at humane endpoints a priori determined as ≥ 20% weight loss from maximum weight recorded. Lung and spleen tissues from moribund mice were evaluated for bacterial burden as described above on the date they met the euthanasia criteria.

### Colony PCR.

Selected colonies were harvested from plated lung homogenates using a disposable loop and transferred into a screwcap tube containing 1.0 mL of TE (10 mM Tris-HCl (pH 8.0) 0.1 mM EDTA). Comparison liquid stock cultures (“bulk DNA”) were also used by transferring 1.0 mL of culture into a screwcap tube. Tubes containing bacterial culture were incubated in a heat block set at maximum (>100 °C) for 10 min to kill bacteria. Samples were cooled and spun briefly. Liquid was drawn up into a 3.0 mL luer-lock syringe barrel and sterile-filtered through a 0.2 μm filter unit. Samples were analyzed by PCR for a previously identified duplication event [23]. We used the JuntA-B forward primer 5′-GTACGTAGCGCATTACGCAGC-3′ and MR3128C-R3 reverse primer 5′-GGAGCTGTTGGCGATGAGTG-3′ to detect the presence of the duplication event at a critical insertion site [23].

### Statistics.

For data comparing HN878.P1 infection in different mouse strains, bacterial burden was assessed between groups using a one-way ANOVA with Tukey’s post-hoc test to correct for multiplicity of comparisons. For all other experiments, bacterial burden was assessed between groups by a two-tailed *t*-test. Survival analyses were plotted on a Kaplan-Meier curve with statistical significance determined by a Mantel-Cox log-rank test. Statistically significant differences are denoted in figures as appropriate using asterisk where * p > 0.05; **p > 0.01; ***p > 0.001 and ****p > 0.0001.
